# Naïve B cells reduce fungal dissemination in *Cryptococcus neoformans* infected Rag1^−/−^ mice

**DOI:** 10.1080/21505594.2017.1370529

**Published:** 2017-10-04

**Authors:** Chad Dufaud, Johanna Rivera, Soma Rohatgi, Liise-anne Pirofski

**Affiliations:** a Department of Immunology and Microbial Sciences, Scripps Research Institute, La Jolla, CA, USA; b Division of Infectious Diseases, Albert Einstein College of Medicine and Montefiore Medical Center, Bronx, NY, USA; c Department of Biotechnology IIT-Roorkee, Uttarakhand, India; d Department of Microbiology and Immunology, Albert Einstein College of Medicine, Bronx, NY, USA

**Keywords:** adoptive transfer, B cell, B-1 B cell, brain infection, *Cryptococcus neoformans*, fungal dissemination, IgM, lung infection, Rag1^−/−^ mouse

## Abstract

IgM and B-1 cell deficient mice exhibit early *C. neoformans* dissemination from lungs to brain, but a definitive role for B cells in conferring resistance to *C. neoformans* dissemination has not been established. To address this question, we developed an intranasal (i.n.) *C. neoformans* infection model in B and T cell deficient Rag1^−/−^ mice and found they also exhibit earlier fungal dissemination and higher brain CFU than wild-type C57Bl/6 (wild-type) mice. To probe the effect of B cells on fungal dissemination, Rag1^−/−^ mice were given splenic (intravenously) or peritoneal (intraperitoneally) B cells from wild-type mice and infected i.n. with *C. neoformans* 7 d later. Mice that received B cells had lung histopathology resembling wild type mice 14 d post-infection, and B-1, not B-2 or T cells in their lungs, and serum and lung IgM and IgG 21 d post-infection. Lung CFU were comparable in wild-type, Rag1^−/−,^ and Rag1^−/−^ mice that received B cells 21 d post-infection, but brain CFU were significantly lower in mice that received B cells than Rag1^−/−^ mice that did not. To determine if natural antibody can promote immunity in our model, we measured alveolar macrophage phagocytosis of *C. neoformans* in Rag1^−/−^ mice treated with naive wild-type IgM-sufficient or sIgM^−/−^ IgM-deficient sera before infection. Compared to IgM-deficient sera, IgM-sufficient sera significantly increased phagocytosis. Our data establish B cells are able to reduce early *C. neoformans* dissemination in mice and suggest natural IgM may be a key mediator of early antifungal immunity in the lungs.

## Introduction

The role that B cells and antibody play in natural resistance to *Cryptococcus neoformans* remains unresolved. In human studies comparing serological responses of HIV-infected (high risk) and HIV-uninfected (low risk) individuals to cryptococcal capsular polysaccharide, glucuronoxylomannan (GXM), levels of GXM-binding IgM were lower in sera of HIV-infected than HIV-uninfected individuals.^1-3^ Similarly, HIV-uninfected solid organ transplant recipients who developed cryptococcosis post-transplant had lower serum levels of pre-transplant GXM-IgM than transplant recipients who did not.^4^ A retrospective study of banked peripheral blood lymphocytes from HIV-infected individuals showed that those who subsequently developed cryptococcosis had lower levels of IgM memory (CD19^+^CD27^+^IgM^+^) B cells than those who did not.^3^ Together, these studies link deficiency of IgM and/or deficiency of memory B cells, a main source of serum IgM,^5^ with risk for human cryptococcosis. Lending credence to this association, IgM memory B cells are depleted in HIV/AIDS.^6^^,^^7^

The aforementioned human studies led our group to seek a better understanding of the roles that B cells and natural IgM may play in resistance to *C. neoformans* in mouse models of B cell and IgM deficiency. Intranasal (i.n.) infection with *C. neoformans* in these models implicated either B-1 cells or IgM in containment of *C. neoformans* in lungs and reduced fungal dissemination to brain. Murine B-1 (CD19^+^CD43^+^IgM^+^) cells are considered a homolog of human IgM memory B cells and mainly produce IgM.^5^^,^^8^^,^^9^ In one model, B-1 cell depletion in *C. neoformans*-infected C57Bl/6 mice resulted in higher lung fungal burdens (CFU), less phagocytosis of *C. neoformans* by alveolar macrophages, and early fungal dissemination than in B-1 cell sufficient mice.^10^ In the foregoing study, adoptive transfer of naïve C57Bl/6 B-1 cells to B-1 cell depleted mice reduced early lung and brain fungal CFU and restored alveolar macrophage phagocytosis to levels comparable to wild-type C57Bl/6. In a different model, *C. neoformans*-infected X-linked immunodeficient (XID) mice, which lack B-1 cells and serum IgM, had higher brain CFU, less alveolar macrophage phagocytosis, and disorganized pulmonary granulomatous responses compared with wild type C57Bl/6.^11^ In another model, mice that lack secreted (serum) IgM (sIgM^−/−^) also had higher brain CFU, disorganized pulmonary pathology, and less alveolar macrophage phagocytosis of *C. neoformans* than wild-type C57Bl/6 mice that was increased to levels comparable to wild-type C57Bl/6 by passive transfer of naïve serum IgM from wild type C57Bl/6 mice.^12^ Although the foregoing studies link either B-1 cells or naive serum IgM to resistance to *C. neoformans* dissemination in mice, abnormalities in B cell development and presence of T cells in sIgM^−/−^ mice^13^ and defects in cellular immunity in XID mice^14^^,^^15^ preclude definitive conclusions. The present study was performed in Rag1^−/−^ mice, which lack B and T cells and antibody, to directly assess the role that B cells may play in resistance to *C. neoformans* dissemination.

## Results

### *C. neoformans* fungal burdens (CFU) in Rag1^−/−^ mice and wild type C57Bl/6 (wild-type) mice

**Figure 1.**
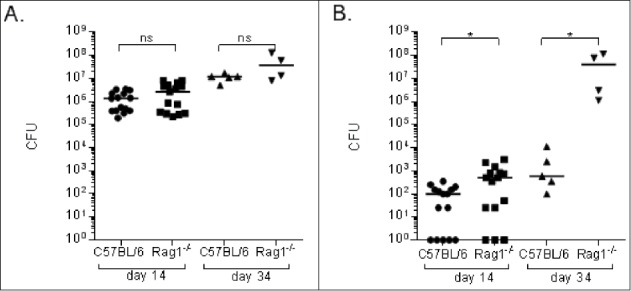
*C. neoformans* fungal burdens in lungs and brains of Rag1^−/−^ and C57Bl/6 (wild type) mice. Fungal burdens, depicted as CFU on the Y axis, in the lungs (A) and brains (B) of Rag1^−/−^ and wild-type mice on the days after infection indicated on the X axis. Each symbol represents one mouse; (A-B) show combined results from 3 separate experiments. Bars represent medians. *p < 0.05, Mann-Whitney test; ns – not significant

*C. neoformans* CFU in lungs and brain of Rag1^−/−^ and wild-type mice were determined at different times after i.n. infection with *C. neoformans*. On day 14 post-infection, CFU in the lungs were similar between Rag1^−/−^ and wild-type mice (Fig. 1a). On day 34 post-infection, lung CFU were higher than on day 14 in both groups, but did not differ significantly between groups. However, brain CFUs of Rag1^−/−^ mice were significantly higher than those of wild-type mice on days 14 and 34 post-infection (Fig. 1b), with median brain CFUs < 10-fold higher in Rag1^−/−^ mice on day 14 and nearly 10^5^ times higher on day 34 after infection (p < 0.05).

### B cells reduce *C. neoformans* dissemination to the brain in Rag1^−/−^ mice

**Figure 2.**
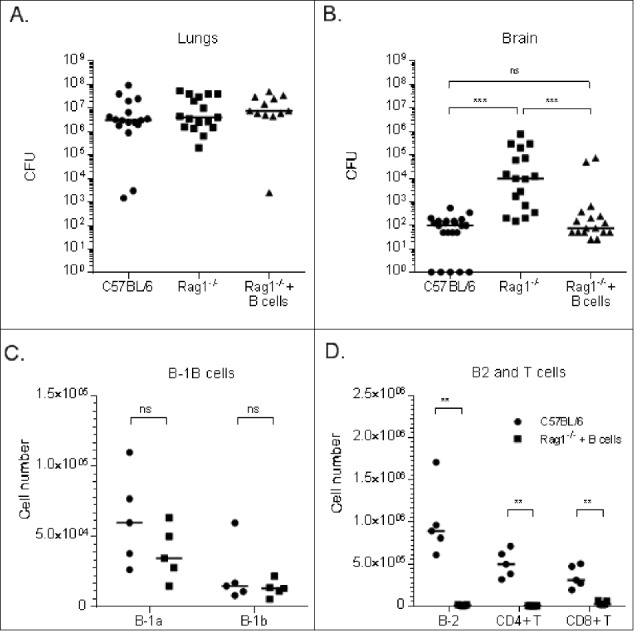
Effect of adoptive transfer of naïve splenic (B)cells to Rag1^−/−^ mice on lung and brain CFU and lymphocyte profiles of *C. neoformans*-infected mice. Lung (A) and (B) brain CFU are depicted 21 d post-infection on the Y axis for wild-type, Rag1^−/−,^ and Rag1^−/−^ (Rag1^−/−^ + B cells) mice that received 10^6^ naïve splenic B cells i.v. one week before infection, depicted on the X axis. Lung lymphocyte numbers of B-1a and B-1b B (C) and B-2 B and T (D) cells determined by flow cytometry are depicted for wild-type and Rag1^−/−^ + B cells mice 21 d post*-C. neoformans* infection (28 d after adoptive transfer). Each symbol represents one mouse; (A-B) present data from 3 separate experiments, (C-D) represent the results of one experiment. (C-D) Figure legend applies to both panels. Bars are medians. **p < 0.01, ***p < 0.001, Kruskall-Wallis test, correcting for multiple comparisons; ns – not significant

The effect of B cells on *C. neoformans* dissemination was determined by adoptive transfer of naïve splenic B cells from wild-type to naïve Rag1^−/−^ mice. Rag1^−/−^ mice received 10^6^ naïve splenic B cells (DAPI^−^ / CD45^+^ / CD19^+^) intravenously (i.v.) 7 d before i.n. infection with *C. neoformans*. On day 21 post-infection (28 d after adoptive transfer), lung CFU of Rag1^−/−^ mice that received naïve splenic B cells were comparable to those of Rag1^−/−^ that did not receive B cells and wild-type mice (Fig. 2a). However, brain CFU of Rag1^−/−^ mice that received B cells were comparable to wild-type mice, and CFU of Rag1^−/−^ that received B cells and wild type mice were each significantly lower than those of Rag1^−/−^ mice that did not receive B cells (Fig. 2b). In a separate experiment, 21 d post-infection, Rag1^−/−^ mice that received B cells had lung B-1a and B-1b cell levels comparable to wild-type mice (Fig. 2c). In contrast to wild-type mice, B-2 and T cells were not detected in lungs of Rag1^−/−^ mice that received B cells (Fig. 2d).

### Total and GXM-binding Ig levels

**Figure 3.**
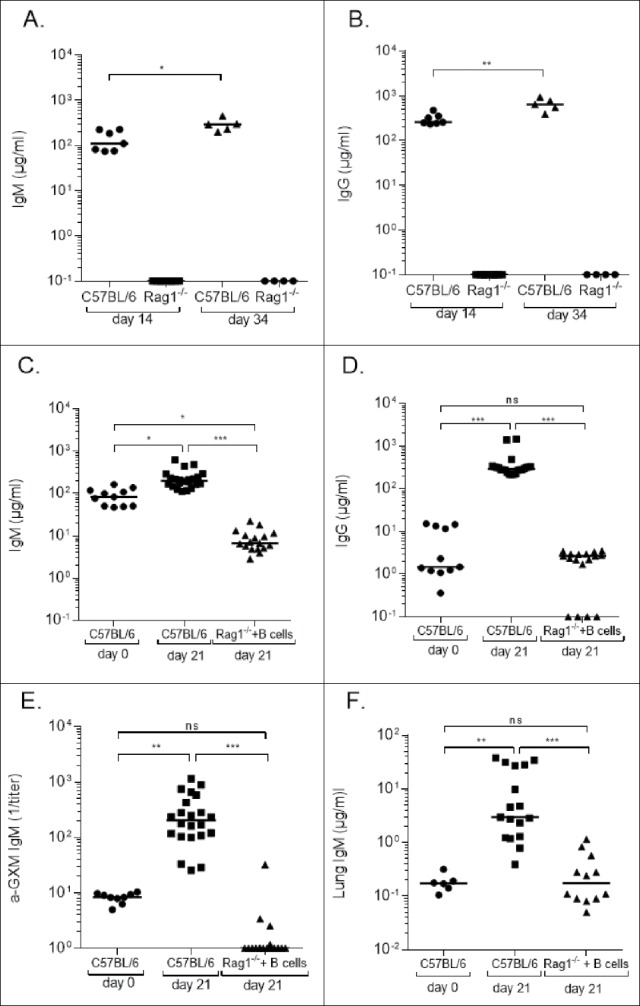
Immunoglobulin (Ig) levels of *Cryptococcus neoformans*-infected C57Bl/6 (wild-type) and Rag1^−/−^ mice. Serum IgM (A, C) and IgG (B, D) levels, depicted on the Y axis, are shown for the mouse groups and times indicated on the X axis. Serum GXM-binding IgM (α GXM IgM) (E) and lung levels of IgM (F) are shown for the groups indicated on the X axis. Each symbol represents one mouse; (C-F) present data from 2 separate experiments. Bars are medians. *p < 0.05, **p < 0.01, **,p < 0.001, ***,p < 0.001, Kruskal-Wallis test, correcting for multiple comparisons; ns – not significant

As expected, Rag1^−/−^ mice had no detectable serum IgM or IgG. Wild-type mice had higher levels of serum IgM and IgG on day 34 than day 14 (Fig. 3a-b), and significantly higher serum IgM on day 21 than day 0 (Fig. 3c). Rag1^−/−^ mice that received B cells had detectable serum IgM 7 d after adoptive transfer (day 0, before infection, not shown) and on day 21 after infection (Fig. 3c), but these levels were significantly lower than those of wild-type mice. Serum IgG was detectable on day 21 in Rag1^−/−^ mice that received B cells, but levels were significantly lower than those of wild-type mice at the same time, though similar to those of naïve wild-type mice (Fig. 3d). Serum IgM and IgG (Fig. S1A) were also detected 14 d post-infection (21 d after B cell transfer) in Rag1^−/−^ mice that received peritoneal B cells.

Serum GXM-binding IgM was detected in wild-type mice on day 0 and significantly higher on day 21 after infection (Fig. 3e). Only a few Rag1^−/−^ mice that received B cells had detectable levels of GXM-binding IgM, and their levels were significantly lower than those in sera of wild type C57Bl/6 mice (Fig. 3e). IgM was also detectable in the lungs of wild-type and Rag1^−/−^ mice that received B cells, but levels were significantly higher in the wild type C57Bl/6 mice (Fig. 3f).Lung IgM (Fig. S1B) and serum GXM-IgM (Fig. S1C) were also detectable 14 d post-infection in mice that received peritoneal B cells.

### Cytokine levels in wild-type and Rag1^−/−^ mice

**Figure 4.**
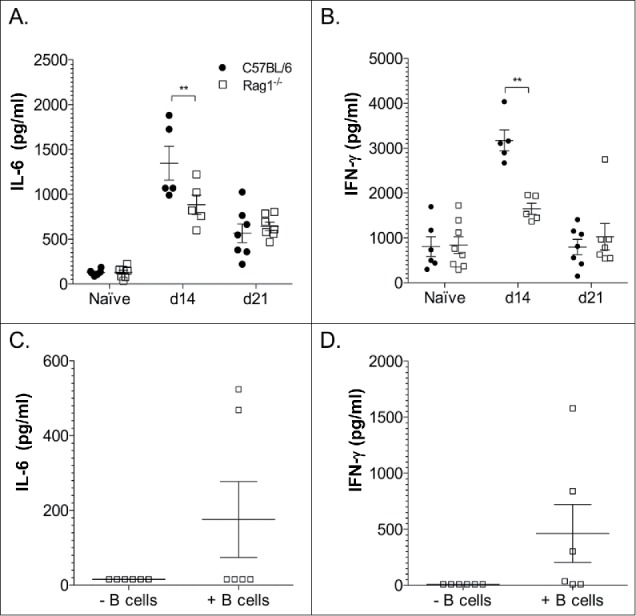
Cytokine levels in the lungs of C57Bl/6 (wild-type) mice and Rag1^−/−^ mice 14 d after *C. neoformans* infection. Interleukin (IL)-6 (A, C) interferon (IFN)-γ (B, D) concentrations are depicted on the Y axis for the mice indicated on the Y axis. The key in panel A refers to all panels in the figure. Open squares represent Rag1-/- mice in Panels (C)and D. Data in Panels C and D, 14 d post-infection. Data from 2 experiments (one in Panels A and B and one in Panels C and D) are shown. Each symbol represents one mouse. Concentrations were determined by ELISA. **p < 0.01, ***p < 0.001, Student's t-test

Rag1^−/−^ mice had lower lung levels of IL-6 and IFNγ than wild type C57Bl/6 mice 14 d post-infection (Fig. 4a, b), but levels did not differ significantly between wild-type and Rag1^−/−^ mice on day 0 or 21 post-infection (Fig. 4a, b). IFN-γ and IL-6 levels were higher in some Rag1^−/−^ mice that received peritoneal B cells than mice that did not (Fig. 4c, d) 14 d post-infection. However, IL-6 was only measureable in 2 of 5 mice and IFN- γ in 3 of 5 mice.

### Effect of naive serum antibody on phagocytosis of *C. neoformans* in Rag1^−/−^ mice

**Figure 5.**
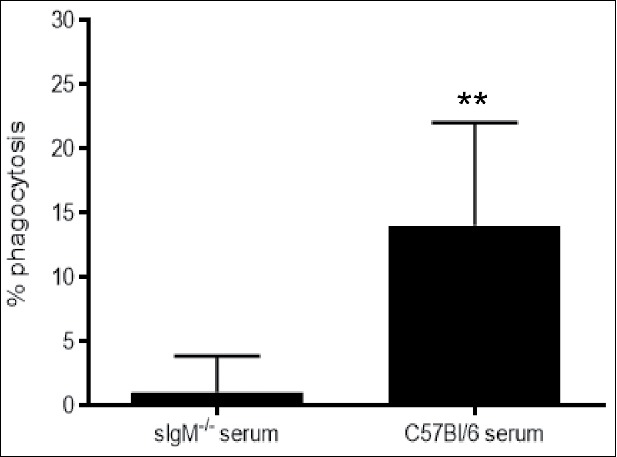
Effect of passive transfer of naïve sera on alveolar macrophage phagocytosis of *Cryptococcus neoformans*-infected Rag1^−/−^ mice. Phagocytosis, measured by the percent of intracellular yeast cells compared with untreated cells, by alveolar macrophages, 24 hrs post-infection is shown on the Y axis for IgM-deficient and IgM-containing IgG-depleted C57Bl/6 serum. Data from 2 independent experiments are shown. **p < 0.01, Student's t-test

Passive transfer of IgG-depleted, IgM-containing serum from wild-type mice promoted significantly more alveolar macrophage phagocytosis and uptake of *C. neoformans* than IgM deficient sera from sIgM^−/−^ mice, 24 hours post-infection (Fig. 5).

### Histopathology

**Figure 6.**
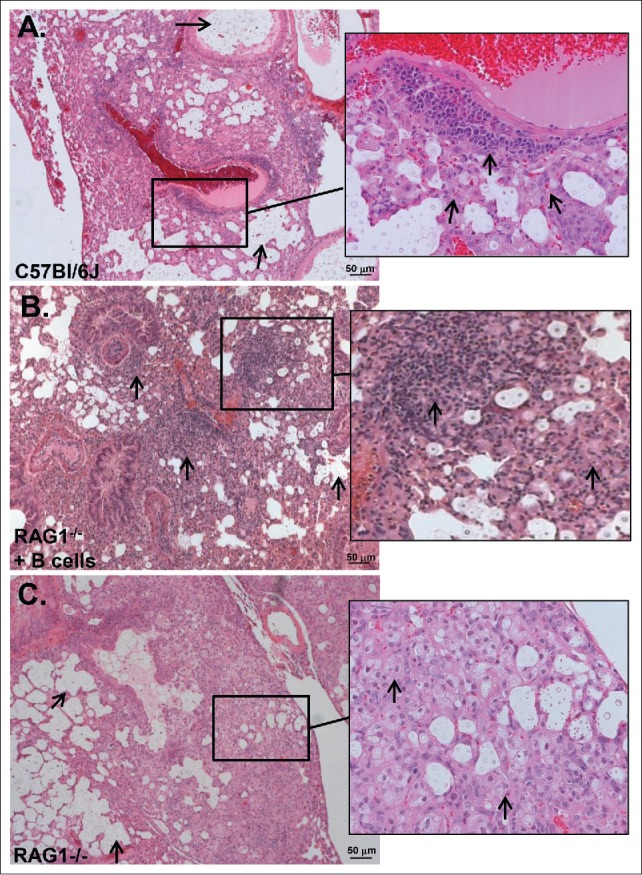
Inflammatory patterns (H&E) of *C. neoforman*s-infected C57Bl/6 (wild-type), Rag1^−/−^ mice that received (B)cells and Rag1^−/−^ mice, 14 d post-infection. A) Wild-type mice. Arrows point to large collections of extracelluar organisms in airspaces. Inset: Organized granulomatous inflammation composed of polymorphonuclear leukocytes, lymphocytes, epithelioid cells and macrophages. Inset: arrows point to lymphocytes and macrophages associated with perivascular inflammation. Five mice were examined. (B) Rag1^−/−^ mice that received peritoneal B cells. Arrows point to organized granulomatous inflammation composed of lymphocytes, polymorphonuclear leukocytes and macrophages associated with perivascular inflammation. There are also large collections of organisms in airspaces. Inset: arrows pointing to lymphocytes and macrophages in cellular infiltrate.  Two mice were examined. (C) Rag1^−/−^ mice. Arrows point to large collections of extracellular organisms in airspaces. Inset: Inflammatory cells are polymorphonuclear leukocytes, epitheloid cells and macrophages as shown by arrows. Five mice were examined. Scale bar = 50 um.

On day 14 post-infection, lungs of wild-type mice exhibited dense inflammation composed primarily of foamy macrophages, polymorphonuclear cells (neutrophils and eosinophils), lymphocytes, and epithelioid cells. In many areas, there were large extracellular collections of *C. neoformans* cells in alveolar spaces. Perivascular cuffs composed of lymphoctytes and polymorphonculear leukocytes were also present (Fig. 6a). Similarly, histopathological analysis of lung sections from Rag1^−/−^ mice that received B cells revealed large areas of organized granulomatous inflammation composed of lymphocytes, polymorphonuclear leukocytes and macrophages (Fig. 6b). In contrast, the lungs of Rag1^−/−^ mice exhibited intense, granulomatous inflammatory responses composed primarily of foamy macrophages and epitheloid cells (Fig. 6c). Rag1^−/−^ mice also had large areas of scant inflammation with large collections of yeast cells in the alveolar space. While the area of lung parenchyma with inflammatory cells was similar between wild-type and Rag1^−/−^ mice, the inflammation was more organized in wild-type and Rag1^−/−^ mice with B cells.

## Discussion

Our data establish that adoptive transfer of B cells from naive wild-type mice to Rag1^−/−^ mice reduces early dissemination of *C. neoformans* to the brain following i.n. infection. While lung CFU of wild-type and Rag1^−/−^ mice were comparable, brain CFU were higher in Rag1^−/−^ mice on days 14 and 34 post-infection. Thus, like B-1 cell deficient XID and secreted IgM deficient sIgM^−/−^ mice,^11^^,^^12^ B and T cell deficient Rag1^−/−^ mice also exhibit an earlier *C. neoformans* dissemination phenotype. Remarkably, we found that adoptive transfer of splenic B cells from naïve wild-type to Rag1^−/−^ mice one week before *C. neoformans* infection resulted in lower brain CFU 21 d post-infection. At this time, lung B-1a and B-1b cell levels were comparable in wild-type and Rag1^−/−^ mice that received B cells, whereas B2, CD4 and CD8 T cells were undetectable in Rag1^−/−^ mice that received B cells. In this regard, our results are consistent with previous work showing B-1 cells migrate to inflammatory foci,^11^^,^^16^^,^^17^ in that only B-1 cells were detected in the lungs of mice that received B cells. Thus, our data indicate that naïve B cells can mediate a reduction in fungal dissemination to the brain in Rag1^−/−^ mice. This confirms and extends results of a previous study which showed that adoptive transfer of naive peritoneal B-1 cells to B-1 cell-depleted C57Bl/6 mice reduced early *C. neoformans* dissemination to brain.^10^

To our knowledge, the role of B cells in the outcome of i.n. *C. neoformans* infection has not been examined previously in Rag1^−/−^ mice, although one study reported that administration of wild-type CD4 cells to Rag1^−/−^ mice resulted in an IRIS-like inflammatory response.^18^ In the foregoing model, brain CFU were lower in CD4 cell-reconstituted Rag1^−/−^ than mice that did not receive CD4 cells,^18^ but the effect of B cells was not evaluated. B and T cells are likely to have complementary effects on antifungal immunity when both are present. However, our data showing higher brain CFU in Rag1^−/−^ mice parallel previous findings in XID, sIgM^−/−^, and B-1 depleted C57Bl/6 mice. Importantly, each of the foregoing mouse strains have T cells, but lack B and/or B1 cells and exhibit more fungal dissemination to the brain than wild type C57Bl/6 mice.^3^^,^^10^^,^^11^

The presence of T cells in sIgM^−/−^ and B-1 depleted C57Bl/6, but not Rag1^−/−^ mice that received B cells in our study, supports the conclusion that B cells can reduce early *C. neoformans* dissemination to the brain in the absence of T cells. In fact, our data are consistent with a previous study in which B cells were required to protect against *C. neoformans* dissemination; adoptive transfer of lymphocytes from *C. neoformans*-immunized B cell-sufficient, but not B cell-deficient mice mediated a reduction in brain CFU in SCID mice.^19^ Our data extend the results of this important study to show that naive B cells reduce brain CFU in mice that lack T as well as B cells. Given that we administered B cells that were sorted by flow cytometry by gating on single CD19+CD45+DAPI- cells, it is very unlikely non-B cells contributed to our results. However, one caveat to our findings is that we cannot exclude the possibility that positive selection of splenic B cells may have activated the cells used for adoptive transfer, although we note that B cell activation occurs *in vivo* upon antigen encounter.

Lung CFU were comparable in Rag1^−/−^ and wild-type mice 21 d post-infection. This parallels previous studies in which lung CFU of XID, sIgM^−/−^, and B-1 depleted C57Bl/6 and wild-type C57Bl/6 mice were comparable.^10-12^ In the current study, lung CFU were modestly, albeit not significantly, higher in Rag1^−/−^ mice than wild-type on day 14 post-infection. This could be due to higher levels of IFN-γ observed in wild-type mice, as IFN-γ is a key mediator of fungal clearance in mice and humans.^20^^,^^21^ Some Rag1^−/−^ mice that received B cells also had higher levels of IFN-γ than mice that did not receive B cells 14 d post-infection, but this was not the case for every mouse and CFU were not determined in the same mice. The lack of evidence for increased levels of inflammatory cytokines in most mice that received B cells suggests that an effect of B cells on the cytokine response may have occurred at an earlier time, or be due to different mediators, e.g. complement,^22^ or different cytokines, or, that more robust B cell reconstitution and/or T cells are needed to produce a pro-inflammatory cytokine response.

Although we cannot link the beneficial effect of B cell administration to Rag1^−/−^ mice to the cytokines we examined, we did find a clear biologic effect in histopathological studies, whereby the lung infiltrates of Rag1^−/−^ mice that received B cells resembled those of wild-type mice. They contained macrophages, lymphocytes, and polymorphonuclear cells, typical of an organized inflammatory response of C57Bl/6 mice to *C. neoformans.*^23^ In contrast, infiltrates of Rag1^−/−^ mice were less organized. Given that lung CFU did not differ between the 3 mouse groups at this time, 14 d post-infection, our data are consistent with the concept that B cells enhance fungal containment by promoting a more histiocytic inflammatory response. Along these lines, antibody-mediated protection against *C. neoformans* was associated with histopathological changes that reduce host damage in the lungs without early fungal clearance.^23^ In fact, in the foregoing study, antibody-mediated immunity in *C. neoformans*-infected mice was dependent on B cells. Although our data did not reveal an effect of B cells on inflammatory cytokines at the time we examined, administration of B cells to Rag1^−/−^ mice led to a histiocytic pattern of inflammation in the lungs similar to that found in wild-type mice in our study, and a reduction in early fungal dissemination without an effect on fungal clearance. These findings give further credence to the hypothesis that B cells may alter the early lung inflammatory response to *C. neoformans* in a beneficial manner and justify future studies of inflammatory and anti-inflammatory mediators at earlier times and/or in situ. As we were not able to obtain lung samples at later times, we do not know how the inflammatory response was affected by B cells over time, or if the inflammatory pattern evolved further in the lungs of Rag1^−/−^ mice.

Our data herein show that B cell administration reduces fungal dissemination to the brain in mice that lack B and T cells. Although we have not yet identified a definitive mechanism to explain the beneficial effect of B cells in our model, our data do support the hypothesis that IgM contributes to antifungal immunity in the lungs. On day 21 post-infection, compared with day 0, wild-type mice exhibited an increase in serum IgM, IgG, and lung IgM, and serum and lung IgM were each detectable in Rag1^−/−^ mice that received B cells 14 and 21 d post-infection, albeit at much lower levels than in wild-type mice. Given that GXM-binding IgM levels were very low or undetectable in most mice that received B cells, the protective effect of B cells in our model may be mediated by natural IgM produced by B-1 cells. B-1-derived IgM binds conserved microbial determinants, including β-glucans.^12^^,^^24^ In support of this concept, it has been shown that, in the early innate immune response, *C. neoformans*-selected B-1 cells secrete laminarin (β-glucan)-binding IgM,^10^ and, that a β-glucan-binding monoclonal antibody can protect mice against *C. neoformans*.^25^ Consistent with a protective role for naïve IgM in our model, naïve IgM-sufficient sera promoted alveolar macrophage phagocytosis of *C. neoformans* more than IgM-deficient sera. Thus, IgM may be the main functional mediator of early macrophage-mediated antifungal activity in the lungs. Nonetheless, we cannot exclude a role for IgG, as it was also present in sera and lungs of wild-type and Rag1^−/−^ mice that received B cells. Thus, further studies are needed to address this question and determine the specificity of antibody that mediates phagocytosis in the lungs of mice that receive B cells.

Our serological findings in wild-type mice and phagocytosis data lead us to hypothesize that administration of B cells to Rag1^−/−^ mice may recapitulate the natural early immune response to *C. neoformans* by promoting antibody-mediated containment of *C. neoformans* in the lungs. Humans are highly resistant to the development of disease with *C. neoformans*, although infection is common,^26^ and mice require very high doses of yeast to develop disseminated disease with the cryptococcal strain used in this study (52D, 24067). Consistent with this concept, our phagocytosis results resemble studies in which administration of naïve serum IgM to IgM-deficient mice, or B-1 cells to B-1 depleted C57Bl/6 mice enhanced alveolar macrophage phagocytosis and/or reduced *C. neoformans* dissemination to the brain.^3^^,^^10^^,^^12^ Although the foregoing studies and our data herein suggest IgM in the lungs may play a key role in reducing *C. neoformans* dissemination, our findings do not rule out a role for other B-1 functions, such as cytokine secretion, phagocytosis by B-1 derived macrophages, and/or direct fungal killing by B-1 cells.^27^^,^^28^ The latter are known properties of B-1 cells. Along these lines, we note that immunity to *C. neoformans* is notable for redundancy and complex interactions among different immune components.^26^ We recognize that our study has limitations, including that its scope and our resources did not uncover the precise mechanism/s by which B cells mediate their beneficial effect in our model. Nonetheless, we submit that our data are sufficient to warrant more work in this area and strongly support the recognition of B cells as a constituent of the early innate response to *C. neoformans*.

## Materials and methods

### Mice

Mice were housed under specific pathogen-free conditions in the Animal Institute at Albert Einstein College of Medicine. Rag knockout (Rag1^−/−^ – B6.129S7-*Rag1^tm1Mom^*/J) and C57BL/6 strains were obtained from Jackson Labs (Bar Harbor, ME). Rag1^−/−^ mice are unable to produce mature B or T cells because they lack V(D)J recombination activation gene RAG1.^29^ C57BL/6 mice were used as wild-type C57Bl/6 for Rag1^−/−^ mice. Secretory IgM deficient (sIgM^−/−^ mice were obtained from John Chan (Einstein). All mouse experiments were conducted with prior approval from the Einstein Animal Care and Use Committee.

### Intranasal infections with *Cryptococcus neoformans*

All experiments were conducted with a serotype D strain of *Cryptococcus neoformans*, 52D (ATCC 24067). This strain has been used extensively for studies of CN pathogenesis and virulence in mice. Aliquots of CN were stored at -80°C in 15% glycerol until needed. CN inocula were grown in Difco Sabouraud dextrose broth (Becton Dickinson, Franklin Lakes, NJ) at 30°C while shaking at 150 rpm for 48h and washed twice in phosphate-buffered saline (PBS, Cellgro, Corning, NY). Viable cells were determined using a hemacytometer (Hausser Scientific, Horsham, PA). Mice were anaesthetized with isoflurane (Halocarbon, River Edge, NJ) and then infected intranasally with 2 × 10^5^ CFU of CN in 20 µl of PBS as described previously.^11^ Inocula were confirmed by plating on Sabouraud dextrose agar plates (BBL, Sparks, MD).

### Collection of blood

Blood was obtained retro-orbitally from anaesthetized mice using heparinized micro-hematocrit tubes (Thermo Fisher Scientific, Waltham, MA). After clotting overnight at 4°C, blood was centrifuged at 8000 x g for 30 minutes, and serum was collected. Serum was centrifuged once more at 13,000 x g for 5 minutes to remove any additional red blood cells and stored at -20°C until analysis.

### Determination of *C. neoformans* fungal burden (CFU)

Lungs and brains were removed from humanely killed mice and homogenized in 2 ml and 1 ml, respectively, of sterile PBS. 10-fold serial dilutions were plated in duplicate on Sabouraud dextrose agar plates. Neat and 1:10 dilutions of freshly extracted blood were similarly plated. Plates were left to grow at 30°C for 48 hours. Visible colonies were counted, and total CFUs per organ and CFUs per ml of blood were determined.

### Total immunoglobulin quantification

Serum and lung levels of immunologlobulin (Ig) were determined by enzyme-linked absorbance assay (ELISA). Briefly, 96-well ELISA plates (Costar, Corning, NY) were coated with 50 µl of 10 µg/ml unlabeled goat anti-mouse IgG or IgM (SouthernBiotech, Birmingham, AL) for 1 hour at 37°C. Wells were emptied and plates were blocked with 200 µl of 1% bovine serum albumin (Sigma-Aldrich, St. Louis, MO) with PBS (1% BSA-PBS) overnight at 4°C. Plates were washed with 0.05% Tween 20 (Sigma-Aldrich) in PBS using an Aquamax 2000 plate washer (Molecular Devices, Bethesda, MD), serum or lung samples were titered on the plates along with IgG and IgM standards (Southern Biotech), after which plates were incubated for 1 hour at 37°C, washed and developed with 10 µg/ml anti-IgG or anti-IgM conjugated with alkaline phosphatase (AP) (SouthernBiotech). Optical densities were measured at 405 nm (OD_405_) using a Sunrise 96-well plate reader and Magellan software (Tecan, Männedorf, Switzerland). After subtracting PBS blanks from OD_405_ values, standard curves for IgG and IgM were developed using total binding site regression (GraphPad Prism, Graphpad, La Jolla, CA), and, using this curve, sample concentrations of Ig were determined by interpolation.

### Determination of GXM-binding Ig

GXM-binding IgM and IgG titers were determined by ELISA as described previously.^12^ Briefly, ELISA plates were coated with 50 µl of 10 µg/ml purified CN52D (24067) GXM in PBS for 3 hours at room temperature. Blocking, sample addition, secondary antibody detection, development and plate reading was performed as described above. Using total binding site regression (Graph Pad Prism), the titer of GXM-specific Ig was defined to be the point at which the regression curve crossed an OD_405_ value of 0.1.

### Lung cytokine quantification

Lung homogenates were centrifuged at 3000 x g for 30 minutes at 4°C to remove debris. Supernatants were collected and stored at -80°C until analyzed. Cytokine levels in lung homogenates were determined using DuoSet ELISA kits according to the manufacturer's instructions (R&D Systems, Minneapolis, MN) as described previously.^30^ Levels of interleukin- (IL) 6 and IFN-γ were determined because of their described previously roles in *C. neoformans* pathogenesis.^12^^,^^31^

### Adoptive transfer of B cells and sera

#### B cells

Spleen cells were collected from naïve wild-type mice after gentle grinding and filtering through a 70 µm strainer as described previously.^11^ Cells were incubated with anti-CD16/CD32 to reduce nonspecific binding, and then labeled with CD45-Alexa 700, and CD19-PE/Cy7 (BD Biosciences, Franklin Lakes, NJ). DAPI was added to identify dead cells. Fluorescence-activated cell sorting (FACS) was used to isolate single cells and quantify live B cells in the lymphocyte population gating on CD19^+^CD45^+^DAPI^−^ cells (Dako Cytomation MoFlo high speed cell sorter (Beckman Coulter, Inc., Indianapolis, IN)). The purity of the sorted B cells was confirmed by flow cytometry, and10^6^ cells were immediately administered i.v. to mice. In some experiments, of peritoneal B cells were isolated and administered i.p. to Rag1^−/−^ mice as described.^11^ Briefly, peritoneal cavity cells were collected from wild-type mice by lavage and B cells were enriched by separating them from adherent peritoneal macrophages after 2 hours of culture in RPMI 1640 supplemented with 10% FBS. Nonadherent B cells were washed, resuspended in PBS and a total of 10^6^ B cells in 0.5 ml PBS were injected i.p. The purity of these cells as DAPI^−^, CD45^+^, CD19^+^, CD3^−^ was confirmed by flow cytometry.

#### Sera

In some experiments, IgM-containing IgG-depleted serum from C57Bl/6 wild type C57Bl/6 mice was administered i.p. to Rag1^−/−^ mice as described.^12^ Briefly, blood samples were collected from C57Bl/6 mice by retro-orbital puncture and serum samples were obtained by centrifugation after allowing the blood samples to clot. The serum was then depleted of IgG using protein G (Pierce) as described, 32 and the amount of IgM was determined by ELISA (IgM:IgG ratio: 9.1:1). For passive transfer experiments, one group of 4 Rag1^−/−^ mice received 300 μl (19.9 μg/ml) of IgG-depleted sera i.p. 1h before *C. neoformans* infection as described above. Another group of 4 Rag1^−/−^ mice received 300 μl of serum collected from whole blood of secretory IgM deficient (sIgM^−/−^) mice. Sera from these mice lacks IgM and contains IgG.^12^ Lung alveolar macrophages were collected from *C. neoformans*-infected Rag1^−/−^ mice by lavage for phagocytosis experiments (see below).

### Analysis of lung leukocyte populations

In a separate experiment, lung leukocyte populations of *C. neoformans*-infected Rag1^−/−^ mice that received B cells were immunophenotyped by flow cytometry to determine the presence of B cell subsets and T cells in their lungs. After the mice were killed, lung leukocytes were isolated and B-1a (CD45^+^CD19^+^IgM^hi^B220^lo^CD5^+^), B-1b (CD45^+^CD19^+^IgM^hi^B220^lo^CD5^−^), B-2 B (CD45^+^CD19^+^IgM^lo^B220^hi^), and CD4^+^ or CD8^+^ T cells (CD45^+^CD3^+^) were identified and quantified as follows: single-cell suspensions from lungs were prepared by treatment with collagenase D and DNase I (Roche, Indianapolis, IN) followed by GentleMACS dissociation (Miltenyi Biotec, Auburn, CA), according to the manufacturer's instructions as described.^11^ After lysing red blood cells with ACK lysis buffer (Gibco, Life Technologies, Carlsbad, CA) and washing with 1% BSA in PBS, cells were stained with following antibodies: CD45-Alexa Fluor 700, CD19-PE/Cy7, IgM-FITC, IgD-Alexa Fluor 647, CD5-PE, B220-PerCP/Cy5.5, CD3-A647, CD4-APC/Cy7, CD8-FITC. Sources of antibodies were BD Biosciences, eBioscience (San Diego, CA), and BioLegend (San Diego, CA). Flow cytometry data was collected using an LSRII flow cytometer (BD Biosciences) and analyzed using FlowJo software (Tree Star, Ashland, OR). Numbers of B1a, B1b, B2 and T cells were determined by calculating the percentage of cell-specific marker events relative to total CD45^+^ events and multiplying by hemocytometer-determined total leukocyte cell counts.

### Phagocytosis experiments

Alveolar macrophage mediated uptake of *C. neoformans* is a central component of host defense against *C. neoformans*. ^31^ *Ex vivo* phagocytosis experiments were performed as described,^33^ with some modifications, to determine the effect of passively transferred sera on alveolar macrophage phagocytosis of *C. neoformans*. To perform these experiments, alveolar macrophages were collected from Rag1^−/−^ mice 24 h after i.n. infection with *C. neoformans* (as described above), which was introduced 1 h after adoptive transfer of either IgM-deficient or IgM-containing sera (as described above). Briefly, the tracheas of killed mice were exposed by a skin incision, and a 20 gauge angiocath (BD Biosciences, Sandy, UT) was advanced into the trachea 3 mm. The lungs were then lavaged 8–10 times through the catheter with sterile calcium and magnesium-free HBSS without Phenol Red (Life Technologies, Grand Island, NY) with 1 mM EGTA (Sigma-Aldrich, St. Louis, MO) using 0.8 ml per wash. The lavage fluids were pooled, and cells were collected by centrifugation. The total cell suspension was collected by centrifugation, and erythrocytes were lysed by resuspending in ice-cold 0.17 M NH_4_Cl and incubating on ice for 10 min. A 10-fold excess of RPMI 1640 solution was then added to make the solution isotonic, the cells were collected by centrifugation. The macrophage monolayer was then washed PBS, fixed with cold absolute methanol, and stained with 1:20 solution of Giemsa. Cell monolayers were examined with a Zeiss Axiobserver microscope equipped with Zeiss Axiocam HRc camera and 40x objective. Cells were counted and the phagocytic index was determined. The phagocytic index is reported as the number of ingested *C. neoformans* divided by the number of macrophages per field.

### Histopathology

Lung samples were prepared from wild-type and Rag1^−/−^ mice, and Rag1^−/−^ mice that received peritoneal B cells on day 14 post-infection. Mice were humanely killed and lungs were removed and fixed in 10% buffered formalin (Fisher, Pittsburgh, PA) for 48–72 hours. Samples were sent to the Einstein Histopathology Facility where they were embedded in paraffin. Sections 5 µm thick were stained by H&E and analyzed under a Zeiss AxioScope II microscope (Carl Zeiss, Thornwood, NY) by a board-certified veterinary pathologist.

### Statistics

Statistical analysis was performed using GraphPad Prism (GraphPad Software, Inc., La Jolla, CA). Tests included parametric or non-parametric tests, depending on the distribution of the data.

## Supplementary Material

KVIR_S_1370529.zip

## Abbreviation

*GXM*: Glucuronoxylomannan
