# Supplementary material for: Naïve B cells reduce fungal dissemination in Cryptococcus neoformans infected Rag1−/− mice
Source: Virulence. 2017 Oct 4;9(1):173–84. doi: 10.1080/21505594.2017.1370529 (PMC5955176; doi:10.1080/21505594.2017.1370529)
Supplement: KVIR_S_1370529.zip [file kvir-09-01-1370529-s001.zip › KVIR_S_1370529.pptx]

## Slide 1
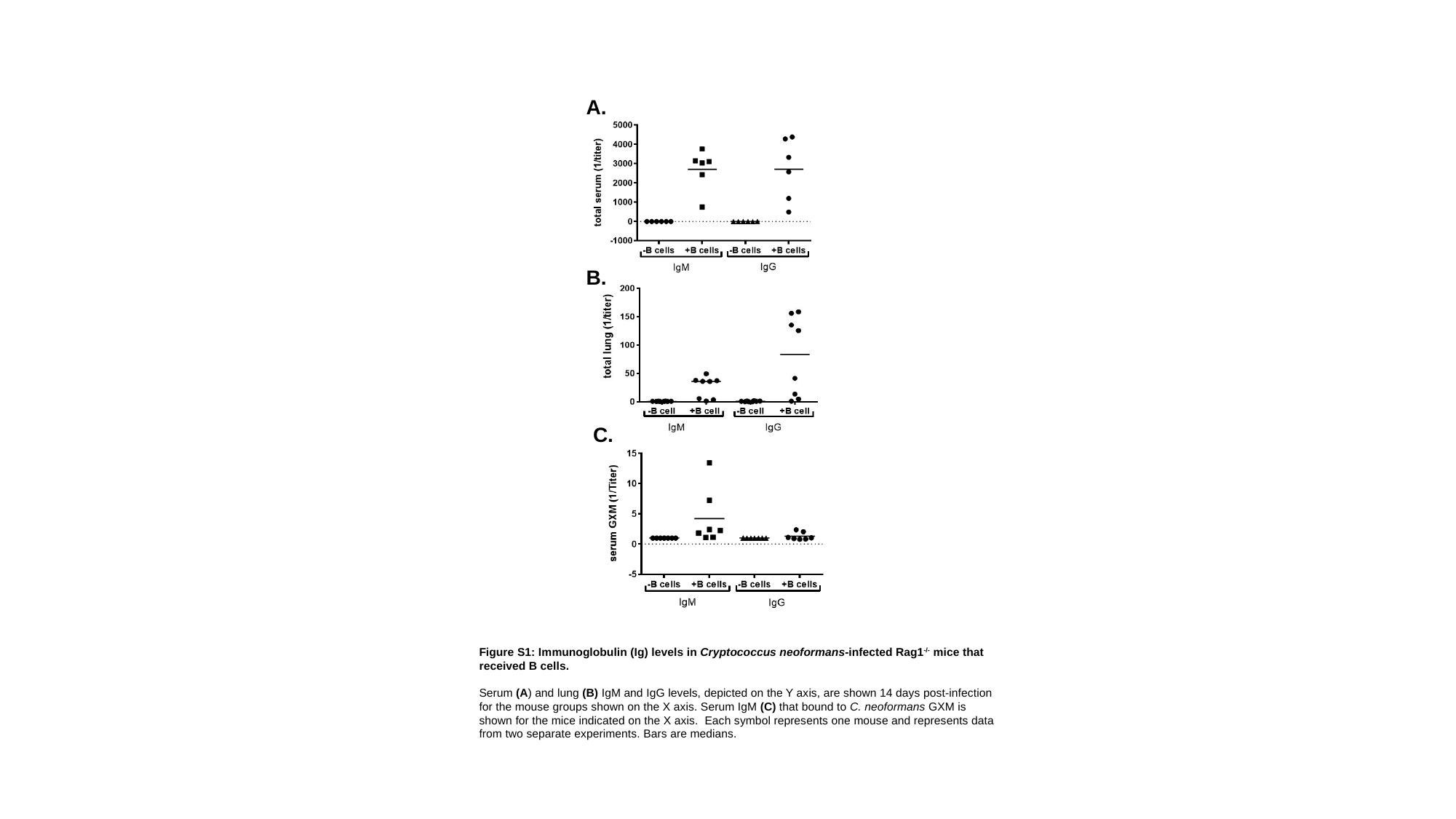

A.
B.
C.
Figure S1: Immunoglobulin (Ig) levels in Cryptococcus neoformans-infected Rag1-/- mice that received B cells.
Serum (A) and lung (B) IgM and IgG levels, depicted on the Y axis, are shown 14 days post-infection for the mouse groups shown on the X axis. Serum IgM (C) that bound to C. neoformans GXM is shown for the mice indicated on the X axis. Each symbol represents one mouse and represents data from two separate experiments. Bars are medians.
